# *Lycium barbarum* Polysaccharide Improves Iron Homeostasis in Spermatocytes and Sertoli Cells via NRF2 to Alleviate DEHP-Induced Male Reproductive Toxicity in Mice

**DOI:** 10.3390/toxics13080677

**Published:** 2025-08-14

**Authors:** Zhen Zhang, Yitong Shang, Hong Yang, Liyang Ding, Yu Deng, Bo Xu, Xufeng Fu

**Affiliations:** Key Laboratory of Fertility Preservation and Maintenance of Ministry of Education, School of Basic Medical Sciences, Ningxia Medical University, Yinchuan 750004, China

**Keywords:** ferroptosis, NRF2, di-(2-ethylhexyl)phthalate, *Lycium barbarum* polysaccharide, Sertoli cell, spermatocytes

## Abstract

Male infertility, as a globally significant reproductive health issue, remains idiopathic in over 40% of cases. Reproductive disorders in males induced by environmental pollutants, such as di(2-ethylhexyl) phthalate (DEHP), have garnered considerable attention in recent years. DEHP induces testicular oxidative stress and ferroptosis via its active metabolite MEHP, thereby leading to spermatogenic dysfunction. *Lycium barbarum* polysaccharide (LBP), a traditional food and medicine homologous substance, exhibits potential antioxidant and reproductive protective properties. However, the underlying mechanism by which LBP intervenes in the toxicity induced by DEHP remains to be elucidated. This study explored the protective effect and molecular mechanism of LBP on DEHP-induced testicular injury through in vivo and in vitro experiments. The result showed that DEHP exposure (150 mg/L in free drinking water for 6 weeks) significantly decreased testicular weight, sperm concentration, and sperm motility in mice, while DEHP exposure induced pathological damage to testicular tissue, as evidenced by cavitation of seminiferous tubules, reduced numbers of spermatocytes, and vacuolar degeneration of Sertoli cells. However, LBP (450 mg/L) treatment significantly reversed testicular damage and sperm parameters. In vitro, MEHP reduced the viability of GC2 cells (spermatocyte cell line) and TM4 cells (Sertoli cell line), and LBP significantly restored cell activity. Mechanistically, exposure to DEHP/MEHP results in iron overload (elevated levels of free Fe^2+^), lipid peroxidation (increased MDA and reduced GSH), and dysregulated expression of key proteins involved in ferroptosis and iron homeostasis within the testis and cells. Furthermore, it was demonstrated that when NRF2 was specifically inhibited by ML385 or silenced via siRNA, the protective effects of LBP were abrogated, thereby validating the critical role of NRF2 in the regulation of iron homeostasis by LBP. In conclusion, LBP mitigates DEHP-induced testicular injury by activating NRF2 to regulate iron homeostasis in Sertoli cells and spermatocytes cells. This study not only offers a potential strategy for the prevention and treatment of male reproductive disorders caused by DEHP exposure, but also underscores the reproductive protective effects and application prospects of LBP in this context.

## 1. Introduction

In recent years, male infertility has constituted over 40% of global infertility cases, emerging as a critical issue in the domain of reproductive health [1]. The role of environmental factors in causing male infertility has gradually attracted attention. The plasticizer di-(2-ethylhexyl) phthalate (DEHP) has emerged as a focal point in the toxicology field. This is attributed to its extensive application in various domains, including food packaging, medical devices, and construction materials, as well as its distinctive reproductive toxicity characteristics [2,3]. As the most extensively utilized phthalate plasticizer globally, DEHP has an annual production volume of several million tons [4]. In industrial applications, DEHP exhibits plasticizing effects by establishing weak intermolecular hydrogen bonds and van der Waals forces with polyvinyl chloride (PVC) materials [5]. However, the non-covalent physical bonding characteristic results in its continuous release into the environment throughout production, use, and disposal, thereby causing multi-media environmental pollution. Environmental monitoring data indicate that DEHP is ubiquitously present in air, water, and soil matrices and can enter the human body via ingestion through the digestive tract, inhalation through the respiratory system, and dermal contact [6,7]. After ingestion, DEHP undergoes hydrolysis to form mono-(2-ethylhexyl) phthalate (MEHP) via the action of non-specific esterases present in the intestines, liver, and blood. Subsequently, MEHP is excreted from the body through renal filtration into the urine [8,9]. Studies have demonstrated that MEHP, the primary active metabolite of DEHP in the body, exhibits a toxicity level more than ten times greater than that of the parent compound and exerts significant targeted toxic effects on reproductive organs, including the testicles [10].

Toxicological studies have confirmed that di(2-ethylhexyl) phthalate (DEHP) exhibits substantial toxic effects on the male reproductive system. Exposure to DEHP has been shown to induce dose-dependent testicular atrophy, structural abnormalities in seminiferous tubules, and histopathological alterations in epididymal tissue in mice [11]. In addition, exposure to DEHP has been shown to result in a reduction in epididymal tail sperm concentration and an elevation in the incidence of sperm malformations. It also significantly impairs sperm motility parameters and increases the sperm DNA fragmentation index (SDF) [12]. However, the specific mechanism by which DEHP causes male reproductive toxicity remains unclear. The testis, serving as the central organ of reproductive function in male mammals, contains seminiferous tubules that function as the fundamental units of spermatogenesis. The seminiferous tubule wall structure is sustained by Sertoli cells and a hierarchically organized arrangement of germ cells at various developmental stages, thereby establishing a dynamic and homeostatic cellular microenvironment [13]. During this process, spermatocytes generate haploid gametes through precisely regulated meiosis [14]. Meanwhile, Sertoli cells supply metabolic support to germ cells via the cytoplasmic synaptic network and are crucial in physiological processes, including the maintenance of the immune microenvironment and the mediation of germ cell migration [15,16,17]. Therefore, we hypothesized that the male reproductive toxicity induced by DEHP may be attributed to its effects on the functions of Sertoli cells and germ cells.

Investigating the prevention and treatment strategies for male reproductive toxicity induced by DEHP holds significant importance in enhancing male reproductive health. *Lycium barbarum* polysaccharide (LBP) is the primary bioactive constituent extracted from the traditional medicinal and edible plant *Lycium barbarum*, and LBP is a complex polysaccharide consisting of arabinose, glucose, galactose, mannose, xylose, and rhamnose [18]. Pharmacological studies have shown that LBP has multiple biological effects, including anti-cancer [19], neuroprotection [20], antioxidation [21], immunoregulation [22], and reproductive system protective effects [23,24]. Numerous studies have confirmed that LBP exhibits protective effects against testicular injury and inhibits male reproductive toxicity. Additionally, LBP has been shown to reverse testicular toxicity and restore sperm motility decreased by Cd exposure [25]. LBP treatment can significantly ameliorate the histopathological alterations in testicular tissue and restore the spermatogenic cell layer disrupted by ionizing radiation [26]. LBP alleviates doxorubicin-induced testicular injury in rats via its antioxidant properties [24]. LBP has the potential to reverse the testicular Sertoli cell injury induced by the herbicide 2,4-dichlorophenoxyacetic acid [27]. Similarly, our previous study has also demonstrated that LBP can alleviate male reproductive toxicity in mice induced by DEHP [28]. Although numerous contemporary studies have demonstrated that LBP exhibits protective effects against testicular injury, the precise mechanism underlying this phenomenon remains to be elucidated.

Ferroptosis is a programmed form of cell death characterized by intracellular iron accumulation, excessive reactive oxygen species production, lipid peroxidation, and disruption of the antioxidant defense system [29]. Studies have demonstrated that ferroptosis plays a critical role in the pathological mechanisms underlying male infertility. This involvement is mediated through dysregulated iron metabolism, oxidative stress, and lipid peroxidation, which collectively result in impaired spermatogenesis and compromised testicular function, ultimately contributing to male infertility [30,31]. Nuclear factor erythrocyte 2-related factor 2 (NRF2) serves as a core transcription factor that regulates cellular iron homeostasis and antioxidant stress responses. Its primary function is to achieve biological effects through the transcriptional activation of downstream target genes [32,33,34]. Previous studies have confirmed that LBP inhibits ferroptosis by activating the NRF2 signaling pathway [28,35]. However, the role and mechanism of LBP in improving ferroptosis of testicular cells by regulating iron homeostasis through NRF2 have not been reported yet.

Therefore, this study proposes the hypothesis that LBP alleviates DEHP-induced testicular toxicity by activating NRF2, thereby regulating iron homeostasis in testicular cells. To further elucidate the mechanism by which LBP ameliorates testicular injury induced by DEHP, a series of in vivo and in vitro experiments were conducted. The results demonstrated that LBP improves iron homeostasis in testicular spermatocytes and supporting cells through the regulation of NRF2. This finding not only elucidates a novel mechanism of DEHP-induced reproductive toxicity but also offers a critical theoretical foundation for the development of strategies to protect the reproductive system using LBP.

## 2. Materials and Methods

### 2.1. Experimental Animals and Treatment

Male C57BL/6 mice (4 weeks of age) were procured from the Animal Center of Ningxia Medical University. All animals were maintained in polypropylene cages with sawdust bedding under specific pathogen-free (SPF) conditions, with a controlled temperature of 23 ± 1 °C, humidity of 55 ± 10%, and a 12 h light/dark cycle. Animals had ad libitum access to sterilized drinking water and standard laboratory chow. At 28 days of age, the mice were randomly divided into three groups (*n* = 10 per group) and administered 0.1% DMSO, 150 mg/L di-(2-ethylhexyl) phthalate (DEHP), or 150 mg/L DEHP + 450 mg/L LBP via their drinking water for 42 consecutive days. Body weights were recorded every five days. All experimental procedures involving animals were conducted in strict compliance with the Guidelines for the Care and Use of Laboratory Animals established by Ningxia Medical University (NXMU-Z010). Following the 42-day treatment period, the animals were euthanized under anesthesia induced by intraperitoneal injection of 1% sodium pentobarbital (0.5 mL/100 g body weight). Blood samples were collected via cardiac puncture, and serum was obtained after centrifugation at 4500 rpm for 10 min. Testes were either snap-frozen in liquid nitrogen for subsequent biochemical analyses or fixed in Bouin’s solution for histopathological examination. Epididymides were harvested for sperm motility analysis.

### 2.2. Evaluation of Sperm Parameters

To assess spermatozoa motility, a computer-assisted semen analysis (CASA) was performed using the TOX IVOS II CASA System (Hamilton Thorne, Beverly, MA, USA). The epididymis was excised and placed into a 1.5 mL Eppendorf tube containing 700 μL of physiological saline. It was then finely minced with ophthalmic scissors to ensure thorough dispersion of spermatozoa and formation of a homogeneous suspension. The suspension was subsequently incubated at 37 °C for 10 min. Following incubation, a sample of the sperm suspension was carefully pipetted onto a counting slide and immediately analyzed. At least three fields of the counting chamber were evaluated per measurement using the animal semen analysis system. Sperm motility was categorized into four classes: rapid progressive (grade I), sluggish progressive (grade II), nonprogressive (grade III), and immotile sperm (grade IV).

### 2.3. Organ Coefficient

The body weights of the mice, as well as the weights of their testes and epididymis, were measured using an analytical balance. Additionally, the testis and epididymis coefficients were calculated. The organ coefficient was evaluated using the following formula: (organ weight/body weight) × 100%.

### 2.4. Hematoxylin-Eosin (H&E) Staining

The testes utilized for histopathological analysis were obtained from mice in different experimental groups and subsequently fixed in Bouin’s solution. Following fixation, the tissues were embedded in paraffin wax, dehydrated in ethanol, and sectioned into 5 μm slices. The sections were stained with hematoxylin and eosin (H&E) to evaluate structural changes in the testicles. After H&E staining, the sections were examined under a digital scanning microscope (Tissue Gnostics, Wien, Austria) to assess testicular morphology and capture representative images.

### 2.5. Immunohistochemical Staining

The testicular tissue was fixed with Bouin’s solution, subsequently embedded in paraffin, and sectioned into 4 μm slices before being mounted onto microscope slides. For antigen retrieval, the dewaxed sections were heated in a 0.01 M citrate buffer at 95 °C for 10 min. Following this, the sections were washed with phosphate-buffered saline (PBS) and treated with 3% hydrogen peroxide (H_2_O_2_) in methanol for 20 min at room temperature to inhibit endogenous peroxidase activity. Subsequently, the sections were coated with 5% bovine serum albumin (BSA) and blocked at room temperature for 30 min. After blocking, the sections were incubated overnight at 4 °C with the primary antibody diluted in PBS (1:200). This was followed by incubation with horseradish peroxidase (HRP)-conjugated secondary antibodies (1:200 in PBS) for 1 h at room temperature. The samples were then washed with PBS and visualized using 3,3′-diaminobenzidine tetrahydrochloride (DAB) as the chromogenic substrate. Finally, the slides were digitally scanned and analyzed using a Tissue Gnostics digital scanning microscope (Tissue Gnostics, Wien, Austria), and the quantitative analysis was performed using ImageJ software (version 1.54p).

### 2.6. Cell Culture and Treatment

The GC2 and TM4 cell lines were procured from Procell Life Science & Technology (Wuhan, China). All experiments were conducted using cells between passages 5 and 15 to ensure consistent biological characteristics. The GC2 spermatocyte cell line and TM4 Sertoli cell line were maintained in Dulbecco’s Modified Eagle Medium (DMEM, HyClone, Beijing, China), supplemented with 10% fetal bovine serum (FBS, Biological Industries, Cromwell, CT, USA) and 1% penicillin-streptomycin solution (Beyotime, Shanghai, China). Cells were cultured under standard conditions at 37 °C with 5% CO_2_ in a humidified incubator. Additionally, the cells were exposed to varying concentrations of MEHP (0–600 μM, Aladdin, Shanghai, China) and/or LBP (0–400 μg/mL, Yuanye Bio-Technology Co., Ltd., Linqu County, China) for durations ranging from 0 to 24 h. In some experiments, cells were co-treated with 10 μM Z-VAD-FMK (Z-VAD), 10 μM Ferrostatin-1, 10 μM Necrostatin-1, or 10 μM N-Acetyl-L-Cysteine (NAC; all reagents sourced from Beyotime, Shanghai, China). The solvent used for all treatments was dimethyl sulfoxide (DMSO), with a final concentration not exceeding 0.1%.

### 2.7. Cell Counting Kit-8 (CCK-8) Analysis

Cell viability was assessed using the CCK-8 assay to determine appropriate concentrations for cellular treatment in this study. GC2 and TM4 cells were seeded in 96-well plates at a density of 8000 cells per well. Following cell attachment, the cells were treated with various concentrations of MEHP (0–600 μM) or LBP (0–400 μg/mL). Additionally, cells were exposed to a combination of 100 μM MEHP and 200 μg/mL LBP for 24 h, either alone or in combination with 10 μM Z-VAD-FMK (z-VAD), 10 μM Ferrostatin-1, 10 μM Necrostatin-1, or 10 μM N-acetylcysteine (NAC). Subsequently, DMEM solution containing 10% CCK-8 was added to each well, and the plates were incubated in the dark for 2 h. Finally, the optical density (OD) was measured at a wavelength of 450 nm using a spectrophotometer to evaluate cell viability.

### 2.8. Malondialdehyde (MDA), Glutathione (GSH), and Total Antioxidant Capacity (T-AOC)

Levels of reduced glutathione (GSH), malondialdehyde (MDA), and total antioxidant capacity (T-AOC) in mouse testicular tissues and GC-2/TM4 cells were quantified using commercial assay kits (Nanjing Jiancheng Bioengineering Institute, Nanjing, China). Specifically: Tissue analysis: Testicular tissues from each experimental group were homogenized, and the supernatant was collected. GSH, MDA, and T-AOC levels were determined strictly following the manufacturer’s protocols. Cellular analysis: Cells were seeded in 6-well plates. After 24 h treatments under specified conditions, monolayers were washed with PBS, digested with 0.25% trypsin, and pelleted by centrifugation at 1000× *g* for 5 min. Cell lysates were then prepared and subjected to quantitative analysis of intracellular GSH, MDA, and T-AOC according to kit specifications. The levels of GSH, MDA, and Fe^2+^ in tissues and cells were quantified using the GSH Assay Kit, Lipid Peroxidation (MDA) Assay Kit, and Fe^2+^ Assay Kit (Nanjing Jiancheng, Nanjing, China). Specifically, testis tissues from each group of mice were harvested, and the concentrations of GSH, MDA, and Fe^2+^ were measured according to the manufacturer’s instructions. In vitro, GC2 and TM4 cells were seeded into 6-well plates and treated under specified conditions for 24 h. Subsequently, the cells were washed with phosphate-buffered saline (PBS), trypsinized, and centrifuged at 1000× *g* for 5 min to obtain the cellular pellet. The intracellular levels of GSH, MDA, and Fe^2+^ were then determined following the protocol provided in the assay kits.

### 2.9. Fe^2+^ Content Determination

Testicular tissue iron content was determined using a Tissue Iron Assay Kit (Nanjing Jiancheng, Nanjing, China), wherein iron is liberated from ferritin-bound stores through acidification and reducing agents, subsequently reduced to ferrous iron (Fe^2+^), and complexed with ferrozine to form a pink chromogen with color intensity proportional to Fe^2+^ concentration. Concurrently, cellular and serum ferrous iron levels were quantified using a Ferrous Ion Assay Kit (Cell Biolabs, Wuhan, China), employing a parallel acid-reduction principle where Fe^2+^ forms a blue complex with Ferene S. For both assays, testicular homogenate supernatants and cell culture supernatants were aliquoted into 96-well plates, and absorbance was measured using a microplate reader per the manufacturer’s protocols. Final concentrations were calculated against standard curves.

### 2.10. RNA Extraction and Quantitative Real-Time PCR (RT-qPCR)

Cells or mice were subjected to the respective treatments, after which total RNA was extracted from GC2 cells, TM4 cells, or testes using TRNzol reagent (Invitrogen, Carlsbad, CA, USA) in accordance with the manufacturer’s protocol. Subsequently, cDNA synthesis was carried out using the Reverse Transcription Kit (Takara, Shiga, Japan). The quality and concentration of RNA and cDNA were assessed by measuring the OD260/OD280 ratio and quantifying the samples using a Nanodrop 2000 spectrophotometer (Thermo, Waltham, MA, USA). Extracted RNA and synthesized cDNA were stored at −80 °C and −20 °C, respectively, for subsequent experiments. Quantitative reverse transcription-polymerase chain reaction (RT-qPCR) analysis was performed using gene-specific primers (Sangon, Shanghai, China) and TBGreen^®^ Premix Ex Taq™ II (Takara, Shiga, Japan) on PCR equipment provided by Bio-Rad Laboratories. The expression level of the housekeeping gene β-actin served as an internal control for normalizing mRNA expression levels. The relative mRNA expression levels were calculated using the 2^−ΔΔCt^ method. The sequences of qRT-PCR primers are summarized in Supplementary Table S1.

### 2.11. Western Blotting Analysis

Proteins extracted from testicular tissue, GC2 cells, and TM4 cells were lysed using RIPA buffer (Beyotime, Shanghai, China). Following centrifugation at 12,000 rpm for 15 min, the supernatant was collected for Western blot analysis. Protein concentrations were determined using the BCA protein assay kit. Subsequently, protein supernatants were subjected to heat denaturation, and 40 μg of total protein from each sample was separated on 10% sodium dodecyl sulfate-polyacrylamide gels and transferred onto polyvinylidene fluoride membranes (PVDF, Millipore, San Salvador, El Salvador). The membranes were blocked with PBST containing 4% skimmed milk for 2 h at room temperature and then incubated with primary antibodies according to the manufacturer’s instructions. After rinsing, the membranes were incubated with horseradish peroxidase (HRP)-conjugated secondary antibodies. Target proteins were detected using ECL reagent (Beyotime, Shanghai, China) in an imaging system. Protein expression levels were quantified by measuring grey scale values using ImageJ software (version 1.54p). Representative images were selected from at least three independent experiments, and relative protein expression levels were normalized to β-actin. Details of the primary antibodies used and their dilution ratios are provided in Table S2.

### 2.12. RNA Interference

Ransfection mixtures were prepared in 1.5 mL tubes as follows: Mixture A: 195 μL DMEM + 5 μL siRNA (siNRF2 for experimental group/siNC for control). Mixture B: 195 μL DMEM + 5 μL Lipofectamine 2000. After gentle mixing by pipetting, both mixtures were incubated at room temperature for 5 min. Mixture A was then slowly added to Mixture B, mixed by pipetting, and incubated for 30 min at room temperature to form transfection complexes. The complexes were added dropwise to each well of 6-well plates containing cells at 60–70% confluence, followed by supplementation with 1.6 mL serum-free DMEM. Plates were gently rocked and transferred to a 37 °C, 5% CO_2_ incubator. After 6 h, the medium was replaced with complete DMEM containing 10% FBS. Cells (GC-2/TM4) were harvested for downstream assays at 24 h post-transfection. Three double strands of RNA fragments—si-275 (sense: 5′-GCAGGACAUGGAUUGAUUUTT-3′; antisense: 5′-AAUCAAAUCCAUGUCCUGCTT-3′), si-793 (sense: 5′-CCGAAUUACAGUGUCUUAATT-3′; antisense: 5′-UUAAGACACUGUAAUUCGGTT-3′), and si-931 (sense: 5′-GCAACUGUGGUCCACAUUUTT-3′; antisense: 5′-AAAUGUGGACCACAGUUGCTT-3′)—of NRF2 were synthesized by GenePharma Co. (Shanghai, China).

### 2.13. Sample Size Determination

The initial study design specified 10 animals per group; however, for analyses of key indicators such as sperm parameters, 6 animals per group were randomly selected using a random number table to satisfy experimental reproducibility requirements and adhere to animal ethics guidelines on sample size optimization. For biochemical assays of GSH, MDA, Fe^2+^, and T-AOC levels, only five animals per group from the same cohort retained sufficient residual testicular tissue after completion of mRNA expression assays and aliquot reservation. This sample size was deemed adequate based on assured sample quality and alignment with commonly reported numbers in similar studies.

### 2.14. Statistical Analysis

Statistical analyses were conducted using GraphPad Prism software (version 9.0). Data normality was initially evaluated via the Shapiro–Wilk test. For comparisons between two groups, an independent-samples t-test was applied. In cases of multiple-group comparisons, one-way ANOVA followed by Tukey’s post hoc test was utilized. All data are expressed as mean ± standard deviation (SD) from no fewer than three independent experiments. Statistical significance was defined as *p* < 0.05, whereas highly significant differences were indicated by *p* < 0.01.

## 3. Results

### 3.1. LBP Can Improve Testicular Injury Induced by DEHP

**Figure 1:**
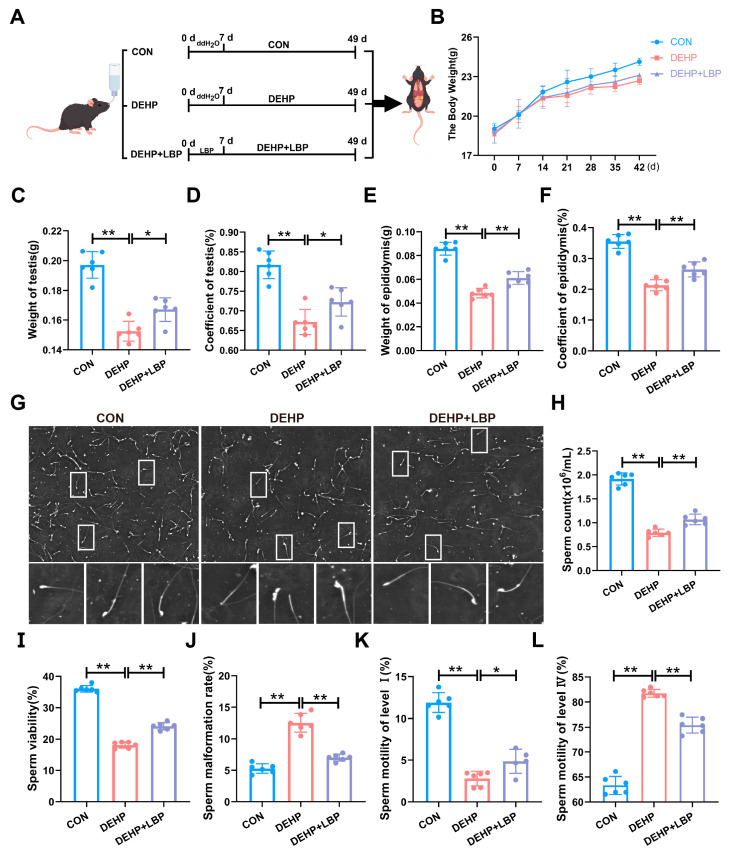
LBP can alleviate testicular injury and sperm quality decline induced by DEHP. (**A**) Mice were treated with DMSO, DEHP (150 mg/L), or DEHP (150 mg/L) + LBP (450 mg/L) via free drinking water for 7 weeks. (**B**) Changes in body weight. (**C**) Testicular weight. (**D**) Testicular coefficient. (**E**) Epididymal weight. (**F**) Epididymal coefficient. (**G**) Sperm morphology. (**H**) Total sperm count. (**I**) Sperm motility. (**J**) Sperm deformity rate. (**K**) Sperm motility grade I: Rapid linear forward movement indicates excellent sperm motility. (**L**) Sperm motility grade IV: Peristaltic movement in place indicates poor sperm motility. Data are expressed as Mean ± SD. * *p* < 0.05, ** *p* < 0.01.

To investigate the efficacy of LBP in ameliorating testicular injury induced by DEHP, 5-week-old male C57BL/6 mice were selected and randomly allocated into three groups following one week of adaptive feeding: (1) DMSO solvent control group; (2) DEHP exposure group (150 mg/L); and (3) DEHP + LBP treatment group (150 mg/L DEHP+ 450 mg/L LBP). The DEHP and LBP were administered via free access to drinking water. The DEHP + LBP group received LBP for one week prior to the start of the experiment, followed by a 6-week administration across all groups (Figure 1A). Subsequently, the body weight changes of mice in each group were measured. Compared with the control group, a significant decrease in body weight was observed in the DEHP exposure group. However, no significant difference was detected between the DEHP + LBP group and the DEHP group (Figure 1B). Compared with the control group, DEHP exposure led to a significant decrease in testicular weight (Figure 1C), testicular coefficient (Figure 1D), epididymal weight (Figure 1E), and epididymal coefficient (Figure 1F), while compared with the DEHP exposure group, these indicators were significantly increased in the DEHP + LBP group. To further investigate the impact of DEHP exposure on spermatogenesis, this study employed the Computer-Aided Semen Analysis System (CASA) to evaluate epididymal sperm parameters. Morphological analysis revealed that sperm morphology in the control group and the DEHP + LBP group was largely normal. In contrast, mice exposed to DEHP exhibited abnormal phenotypes, including head deformities, body distortions, and flagellar abnormalities (Figure 1G). Furthermore, the sperm concentration in the DEHP exposure group was significantly reduced compared to the control group, whereas the sperm count in the DEHP + LBP group exhibited a significant increase relative to the DEHP group (Figure 1H). Analysis of sperm motility parameters revealed that, in comparison to the control group, the sperm motility rate and the proportions of grade I and grade II motile sperm were significantly reduced in the DEHP group. Conversely, the sperm deformity rate and the proportions of grade III and grade IV motile sperm were markedly elevated. However, these adverse effects on sperm parameters were significantly mitigated in the LBP treatment group (Figure 1I–L and Figure S1A,B). These results suggest that LBP is capable of effectively mitigating testicular tissue damage and the decline in sperm quality induced by DEHP exposure.

### 3.2. LBP Can Improve the Functional Damage of Mouse Testicular Spermatogenic Cells and Sertoli Cells Induced by DEHP

**Figure 2:**
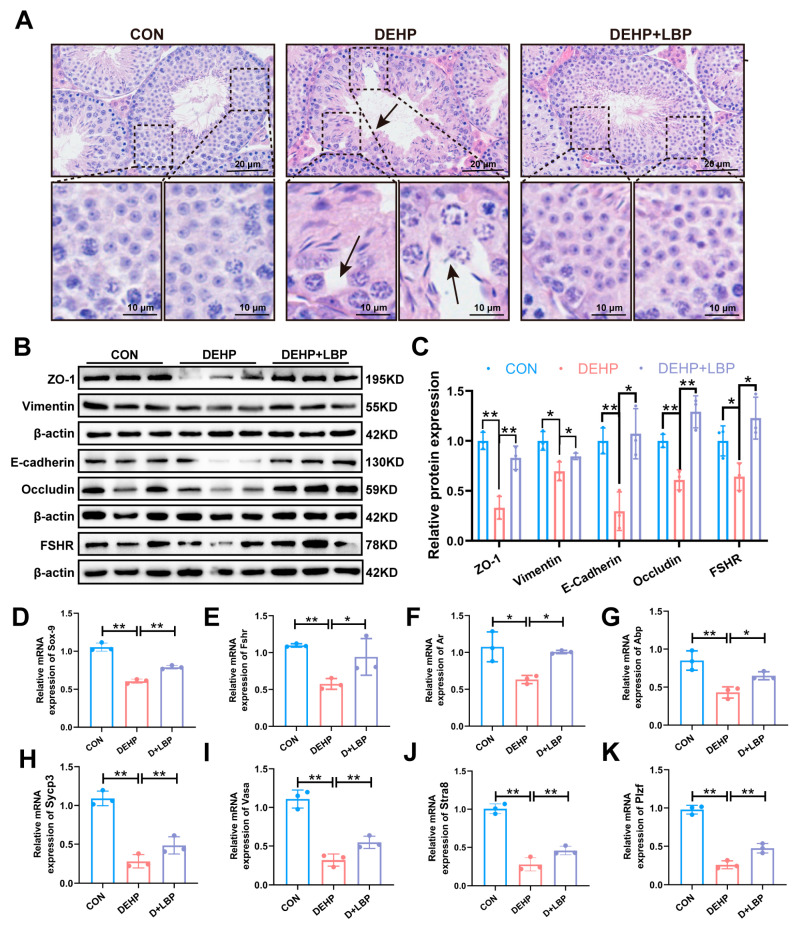
LBP can mitigate the structural and functional damage of testicular spermatocytes and Sertoli cells induced by DEHP. (**A**) Histopathological analysis. Scale bars: 20 μm, 10 μm. (**B**) Expression of ZO-1, Vimentin, E-cadherin, Occludin, and FSHR, which are associated with the structure and function of Sertoli cells in testicular tissue (*n* = 3). (**C**) Quantitative analysis in (**B**). mRNA expression of genes *Sox9* (**D**), *Fshr* (**E**), *Ar* (**F**), and *Abp* (**G**) related to Sertoli cell functions in testicular tissue (*n* = 3). mRNA expression of genes *Sycp3* (**H**), *Vasa* (**I**), *Stra8* (**J**), and *Plzf* (**K**) related to spermatocyte function in testicular tissue (*n* = 3). Data are expressed as mean ± SD. * *p* < 0.05, ** *p* < 0.01.

Further, histopathological analysis was performed on the testes of mice. The results indicated that vacuolation was evident in the lumen of testicular tissues in the DEHP-exposed group. Spermatogenic cells exhibited loose arrangement, and the number of spermatocytes was significantly diminished. Additionally, the near-lumen layer contained a reduced number of spermatozoa, and vacuolation occurred within the Sertoli cells. In contrast, in both the control group and the LBP-treated group, the spermatogenic epithelial structure of the seminiferous tubules in the mouse testes remained relatively intact. Spermatogenic cells were tightly arranged at all developmental stages, and morphologically regular spermatozoa were uniformly distributed within the lumen. Furthermore, the LBP treatment resulted in a notable reduction in the vacuolation of Sertoli cells and an improvement in the number of spermatocytes (Figure 2A). This result suggests that LBP may have improved testicular damage caused by DEHP by enhancing the functions of spermatocyte and Sertoli cells. Therefore, the expression changes of genes associated with the structure and function of Sertoli cells were further investigated. Western blot analysis revealed that following DEHP exposure, the protein expressions of key molecules related to Sertoli cell function, including tight junction protein-1 (ZO-1), Vimentin, E-cadherin, Occludin, and follicle-stimulating hormone receptor (FSHR), were significantly down-regulated. Supplementation with LBP was found to significantly reverse these alterations in gene expression levels (Figure 2B,C). Additionally, a comparable trend was observed upon qRT-PCR analysis. Treatment with LBP also significantly restored the abnormal expression of critical genes such as SRY-box transcription factor 9 (*Sox9*, Figure 2D), follicle-stimulating hormone receptor (*Fshr*, Figure 2E), androgen receptor (*Ar*) (Figure 2F), and androgen-binding protein (*Abp*, Figure 2G). In the seminiferous tubules, Sertoli cells provide structural microenvironments and energy support for spermatocytes by constructing the blood–testis barrier and secreting metabolic substrates [36].

The function of spermatocytes was further detected. The results of qRT-PCR showed that after DEHP exposure, the markers of spermatocyte meiosis, such as synaptic complex protein 3 (*Sycp3*, Figure 2H), DEAD-box helicase 4 (*Vasa*, Figure 2I), Stimulated by retinoic acid gene (*Stra8*, Figure 2J), and promyelocytic leukemia zinc finger (*Plzf*, Figure 2K), were significantly down-regulated, and after LBP treatment, the change levels of the above-mentioned genes can be significantly reversed. These results suggest that LBP may resist the testicular function damage induced by DEHP exposure by improving the functions of testicular Sertoli cells and spermatocytes.

### 3.3. LBP Can Ameliorate Ferroptosis of Mouse Testicular Tissue Induced by DEHP

**Figure 3:**
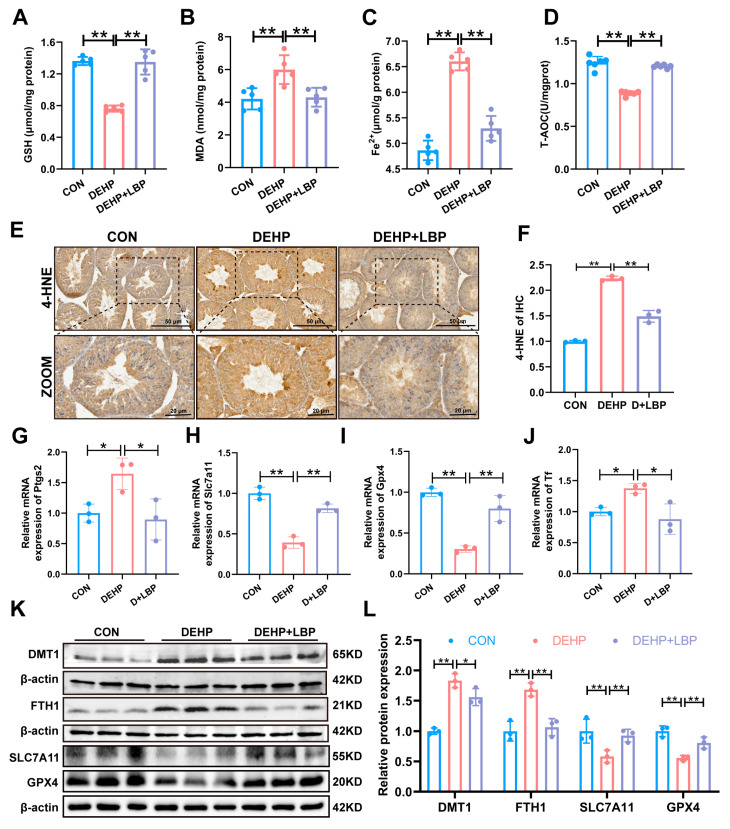
LBP can ameliorate DEHP-induced ferroptosis in mouse testis. (**A**) GSH, (**B**) MDA, (**C**) Fe^2+^, and (**D**) T-AOC levels in testicular tissue. (**E**) Expression of 4-HNE in testicular tissue detected by immunohistochemistry. Scale bars: 50 μm, 20 μm. (**F**) Quantitative analysis in (**E**). mRNA expression levels of ferroptosis-related genes *Ptgs2* (**G**), *Slc7a11* (**H**) and *Gpx4* (**I**) and iron homeostasis-related gene *Tf* (**J**) in testicular tissue. (**K**) Expression of ferroptosis-related proteins DMT1, FTH1, SLC7A11, and GPX4 in testicular tissue. (**L**) Quantitative analysis in (**K**) (*n* = 3). Data are presented as mean ± SD. * *p* < 0.05, ** *p* < 0.01.

Previous studies have shown that DEHP can cause ferroptosis in mouse testicular cells [11,37]. Moreover, our previous study has also shown that LBP can restore the testicular toxicity caused by DEHP [28]. Therefore, we propose the hypothesis that LBP can regulate ferroptosis in the testis and thereby improve DEHP-induced testicular injury. Ferroptosis is a form of regulated cell death characterized by the intracellular accumulation of free iron and lipid peroxides [38]. By detecting the core indicators of ferroptosis, the results showed that compared with the control group, DEHP exposure led to a decrease in total antioxidant capacity (T-AOC) and glutathione (GSH) levels in testicular tissue, accompanied by the accumulation of malondialdehyde (MDA) and overload of free Fe^2+^. However, supplementation with LBP could significantly reverse the above oxidative stress damage and the occurrence of ferroptosis (Figure 3A–D). Furthermore, immunohistochemical detection of the oxidative stress and ferroptosis marker 4-hydroxynonenal (4-HNE) confirmed that LBP could effectively inhibit the oxidative stress injury and ferroptosis induced by DEHP (Figure 3E,F). The results of qRT-PCR showed that DEHP exposure led to a down-regulation of mRNA levels of the key ferroptosis suppressors glutathione peroxidase 4 (GPX4) and solute carrier family 7 member 11 (SLC7A11), and an up-regulation of mRNA levels of the ferroptosis-promoting factors prostaglandin-endoperoxide synthase 2 (PTGS2) and transferrin (TF). Supplementation with LBP could significantly reverse the abnormal expression of these genes (Figure 3G–J). Western blotting analysis further confirmed that DEHP exposure significantly decreased the protein expression levels of GPX4 and SLC7A11, while up-regulating the expression of divalent metal transporter 1 (DMT1) and iron storage protein (FTH1). After supplementation with LBP, the expression of these proteins was significantly reversed (Figure 3K,L). These results indicate that LBP improves ferroptosis of testicular spermatocytes and Sertoli cells by inhibiting DEHP-induced lipid peroxidation accumulation and iron overload.

### 3.4. LBP Can Improve the Damage of Spermatocytes and Sertoli Cells Caused by MEHP

**Figure 4:**
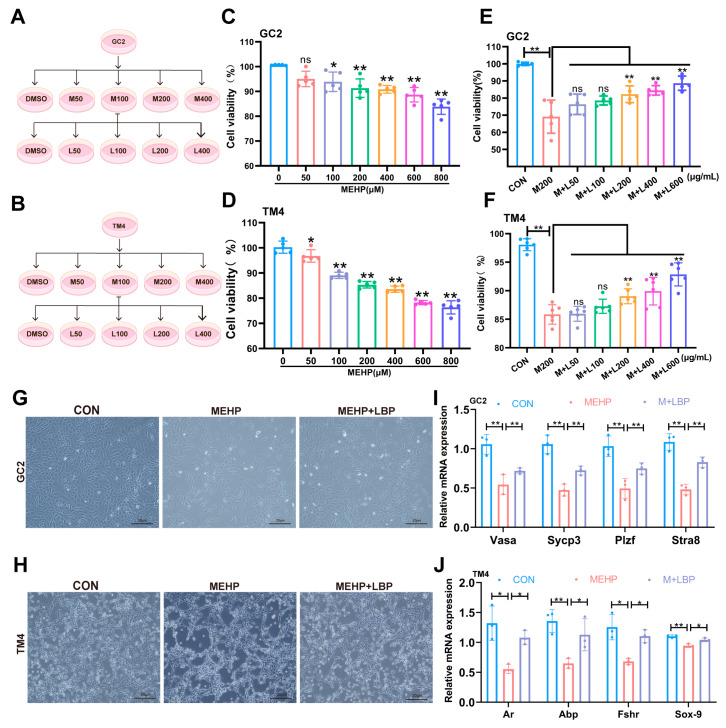
LBP can improve the decreased viability and functional damage of GC2 cells and TM4 cells induced by MEHP. Schematic diagram of the treatment concentrations of MEHP and LBP on GC2 cells (**A**) and TM4 cells (**B**). Concentration screening of the effect of MEHP on the viability of GC2 cells (**C**) and TM4 cells (**D**). Cell viability of LBP in improving the GC2 cells (**E**) and TM4 cells (**F**) caused by MEHP. LBP improves the morphology of GC2 cells (**G**) and TM4 cells (**H**) caused by MEHP. (**I**) mRNA levels expression of GC2 cell function-related genes *Sycp3*, *Stra8*, *Vasa*, and *Plzf* after treatment with LBP and MEHP. (**J**) mRNA levels expression of TM4 cell function-related genes *Fshr*, *Sox-9*, *Abp* and *Ar*, after treatment with LBP and MEHP. Data are presented as mean ± SD. * *p* < 0.05, ** *p* < 0.01, “ns” indicates non-significant differences.

To systematically evaluate the protective effect of LBP on DEHP-induced testicular cells, this study established an in vitro injury model based on the active metabolite MEHP of DEHP: using mouse spermatocyte cell line (GC2) and supporting cell line (TM4) (Figure 4A,B), the dose-dependent analysis was conducted through the CCK-8 cell assay to determine the optimal concentration of MEHP and the effective concentration of LBP. The results showed that when GC2 cells were exposed to 200 μM MEHP for 24 h, their cell viability was significantly reduced. Meanwhile, TM4 cells showed a significant decrease in viability after being treated with 100 μM MEHP for 24 h (Figure 4C,D). Notably, supplementing 200 μg/mL of LBP could significantly restore the cell viability of GC2 cells exposed to 200 μM MEHP and TM4 cells exposed to 100 μM MEHP (Figure 4E,F). Morphological assessment of cells further confirmed that 200 μg/mL LBP could effectively improve the morphological abnormalities of GC2 cells and TM4 cells induced by MEHP (Figure 4G,H). Furthermore, the relevant functional indicators of GC2 cells and TM4 cells were analyzed by qRT-PCR. The results showed that the expression levels of functional marker genes in GC2 cells (*Sycp3*, *Atra8*, *Vasa*, and *Plzf*) and function-related genes in TM4 cells (*Fshr*, *Sox-9*, *Abp* and *Ar*) were significantly down-regulated after MEHP exposure. Moreover, LBP supplementation could significantly reverse the abnormal expression of these genes (Figure 4I,J), which was consistent with the results at the animal level. These results indicate that LBP can improve the functional damage of spermatocytes and Sertoli cells induced by MEHP.

### 3.5. LBP Resists MEHP-Induced Ferroptosis in GC2 Cells and TM4 Cells

**Figure 5:**
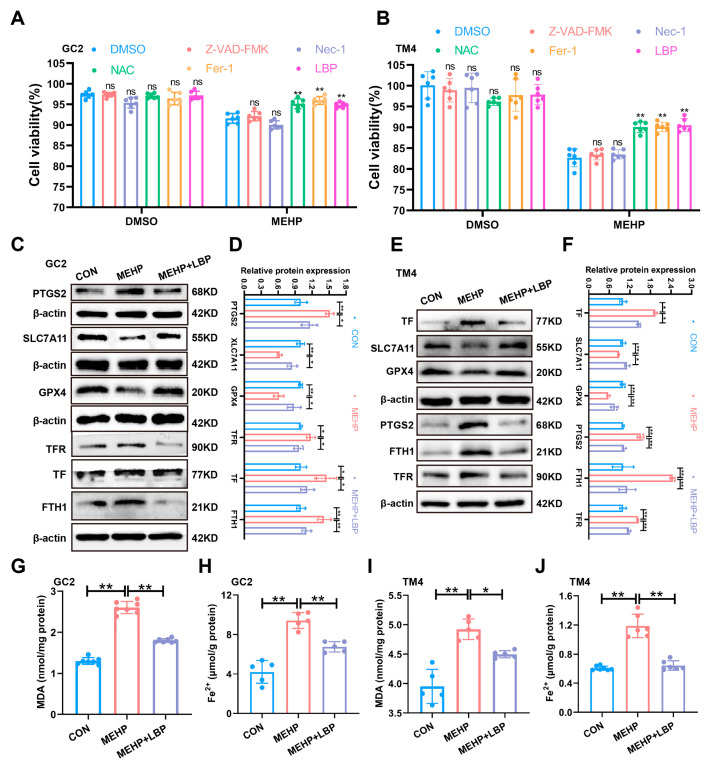
LBP resists ferroptosis in GC2 cells and TM4 cells induced by MEHP. The viability of apoptosis inhibitor (Z-VAD-FMK), necroptosis inhibitor (Nec-1), ferroptosis inhibitor (Fer-1), antioxidant (NAC), and LBP treatment in GC2 cells (**A**) and TM4 cells (**B**). The expression of ferroptosis-related proteins (PTGS2, SLC7A11, and GPX4) and iron homeostasis-related proteins (TFR, TF, and FTH1) in GC2 cells (**C**) and TM4 cells (**E**) after LBP and MEHP treatment. (**D**) Quantitative analysis in (**C**). (**F**) Quantitative analysis in (**E**). The MDA levels (**G**,**I**) and Fe^2+^ (**H**,**J**) levels of GC2 cells and TM4 cells were treated with MEHP and LBP. Data are presented as mean ± SD. * *p* < 0.05, ** *p* < 0.01, “ns” indicates non-significant differences.

To clarify the role of LBP in alleviating MEHP-induced ferroptosis in GC2 cells and TM4 cells, this study treated GC2 cells and TM4 cells with apoptosis inhibitor (Benzyloxycarbonyl-Val-Ala-Asp (OMe)-fluoromethylketone, Z-VAD-FMK), necrosis inhibitor (Necrostatin-1, Nec-1), ferroptosis inhibitor (Ferrostatin-1, Fer-1), antioxidant (N-acetylcysteine, NAC), and LBP, separately. The CCK-8 assay results indicated that Fer-1, NAC, and LBP could significantly reverse the decline in cell viability of GC2 and TM4 cells caused by MEHP, while Z-VAD-FMK and Nec-1 failed to reverse this effect (Figure 5A,B). This indicates that LBP may restore cell survival rate by inhibiting MEHP-induced ferroptosis. Western Blotting results indicated that the expression of SLC7A11 and GPX4 proteins in GC2 cells and TM4 cells was significantly down-regulated after MEHP exposure, while the expression of PTGS2, TF, TFR, and FTH1 was significantly up-regulated, and LBP treatment could effectively reverse these changes (Figure 5C,F). Furthermore, MEHP exposure significantly increased the level of MDA, the end product of lipid peroxidation, in GC2 cells and TM4 cells, while LBP treatment could reverse the effects caused by MEHP (Figure 5G,I). Meanwhile, LBP could also reverse the increase in Fe^2+^ in GC2 cells and TM4 cells caused by MEHP (Figure 5H,J). These results indicate that LBP exerts a protective effect by improving the iron homeostasis imbalance in GC2 spermatocytes and TM4 cells caused by MEHP.

### 3.6. LBP Resists DEHP-Induced Ferroptosis of Testicular Spermatocytes and Sertoli Cells by Activating NRF2

**Figure 6:**
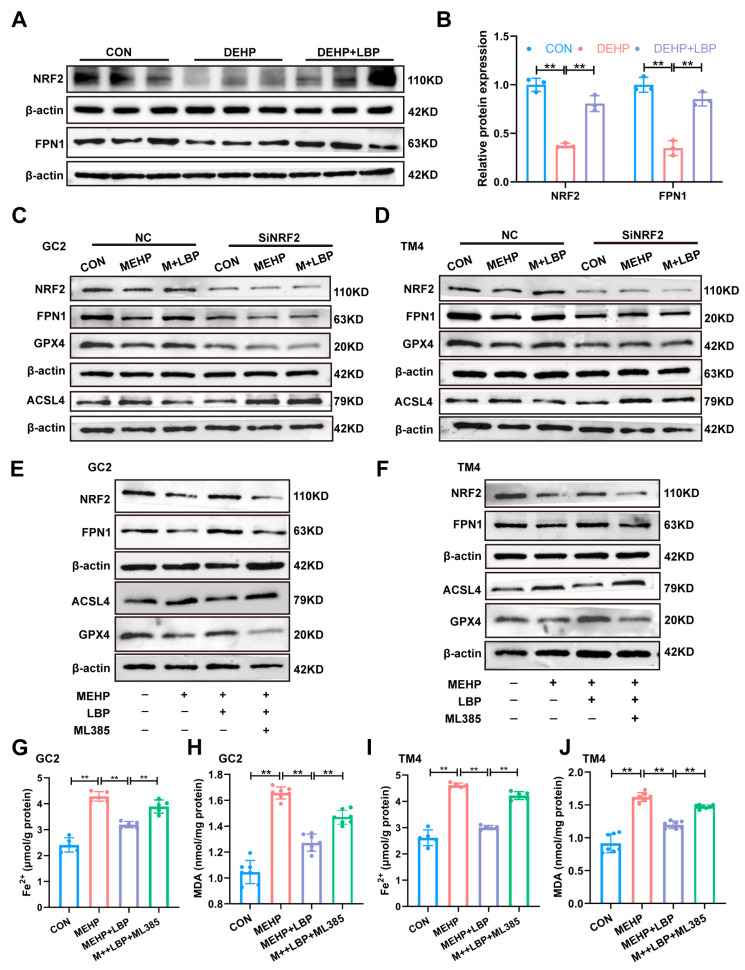
LBP alleviates MEHP-induced ferroptosis via NRF2. (**A**) Protein expression of NRF2 and FPN1 in mouse testicular tissues. (**B**) Quantitative analysis in (**A**) (*n* = 3). Protein expression of NRF2, FPN1, GPX4, and ACSL4 in GC2 cells (**C**) and TM4 cells (**D**) after SiRNA of NRF2, as well as MEHP and LBP treatment. Protein expression of NRF2, FPN1, GPX4, and ACSL4 in GC2 cells (**E**) and TM4 cells (**F**) after treatment with ML385, MEHP, and LBP. Fe^2+^ (**G**,**I**) and MDA levels (**H**,**J**) in GC2 cells and TM4 cells after treatment with ML385, MEHP, and LBP. Data are presented as mean ± SD. ** *p* < 0.01.

Our previous study indicates that NRF2 plays a crucial role in maintaining iron homeostasis [39]. Moreover, as a key transcription factor against ferroptosis, NRF2 directly regulates the expression of genes related to iron homeostasis (such as FPN1, SLC40A1, and GPX4), thereby reducing iron overload and lipid peroxidation and inhibiting ferroptosis [40]. Therefore, the expression of NRF2 and the key iron homeostasis protein FPN1 in the testicular tissues of the control group, DEHP group, and DEHP + LBP group was detected by Western blotting. The results showed that the expression of NRF2 and FPN1 in the testicular tissues was significantly decreased after DEHP exposure, while the supplementation of LBP could significantly reverse the expression levels of NRF2 and FPN1 (Figure 6A,B). These results suggest that LBP may maintain intracellular iron homeostasis by activating NRF2 expression. To further elucidate the role of NRF2 in LBP-mediated alleviation of MEHP-induced ferroptosis, siRNA interference was conducted on GC2 cells and TM4 cells in vitro. The results demonstrated that upon NRF2 interference, its expression was significantly down-regulated in both GC2 and TM4 cells, and LBP failed to reverse the MeHP-induced down-regulation of NRF2 expression. Moreover, after NRF2 interference, LBP was also unable to restore the expressions of key ferroptosis-related proteins GPX4 and ACSL4, as well as the critical iron homeostasis protein FPN1 (Figure 6C,D and Figure S2E–L). This phenomenon was further confirmed by the NRF2 inhibitor ML385. After the addition of ML385, LBP was unable to reverse the effects of MEHP on the expression of NRF2, FPN1, GPX4, and ACSL4. These studies suggest that LBP may improve iron homeostasis by activating NRF2 expression, thereby alleviating ferroptosis in GC2 cells and TM4 cells caused by MEHP. Furthermore, after interfering with NRF2, the ameliorative effects of LBP on the elevation of MDA and Fe^2+^ overload caused by MEHP were significantly inhibited (Figure S2M–T). These results indicate that LBP resists MEHP-induced ferroptosis in GC2 cells and TM4 cells by activating NRF2 and restoring intracellular iron homeostasis.

## 4. Discussion

In this study, in vivo experiments revealed that DEHP-induced damage to Sertoli cells and spermatocytes contributes to testicular toxicity, while LBP can mitigate this adverse effect. In vitro, TM4 and GC2 cells were treated with MEHP, the primary in vivo metabolite of DEHP. The results demonstrated that MEHP disrupts iron homeostasis in both cell types. Furthermore, LBP treatment restored iron homeostasis in MEHP-exposed TM4 and GC2 cells by modulating the NRF2 pathway. This suggests that LBP improves the testicular toxicity caused by DEHP through regulating the iron homeostasis of Sertoli cells and spermatocytes.

Numerous studies have consistently confirmed that DEHP, as a phthalate plasticizer widely used in the plastic industry, has significant toxic effects on the male reproductive system of mammals [41,42]. Studies on toxicity mechanisms have demonstrated that DEHP disrupts the homeostasis of the oxidoreductase–antioxidant system via its metabolites, inducing oxidative stress responses in testicular tissue and consequently resulting in a range of pathological alterations, including abnormal structures of seminiferous tubules, dysfunction of spermatocytes, and impairment of Sertoli cells [43,44].

*Lycium barbarum* polysaccharide (LBP) is a water-soluble plant polysaccharide extracted from *Lycium barbarum*. As a natural product with a variety of beneficial effects, a large number of studies have demonstrated that LBP has antioxidant capacity and protects the reproductive system [45,46]. This plant polysaccharide exhibits significant bioactivity and is readily soluble in water. On the other hand, the DEHP used in our study is a lipophilic compound characterized by a benzene ring diester structure with two ester functional groups; it is also non-volatile at room temperature [9,47,48,49]. Due to their respective highly hydrophobic and hydrophilic properties, we do not believe that DEHP and LBP can interact when present simultaneously in drinking water. We administered test substances via ad libitum drinking water to eliminate gavage-induced physical injuries and stress responses, thereby ensuring strict compliance with animal welfare guidelines. Consequently, in this study, mice were continuously exposed to 150 mg/L DEHP via their drinking water. To observe the protective effects of LBP, 450 mg/L LBP was added to the drinking water and provided to the mice starting one week prior to the initiation of DEHP exposure. Subsequently, throughout the entire experimental period, mice received drinking water containing both DEHP and LBP simultaneously. This protocol of preemptive LBP supplementation has been utilized in several previous studies [50,51]. Simultaneously, the C57BL/6 mouse strain used in this study is obesity-prone and exhibits higher baseline levels of oxidative stress. This characteristic may confer heightened sensitivity to the metabolic regulatory and antioxidant effects of LBP, thereby facilitating the observation of its protective effects [52,53,54].

In toxicology research, low and high doses of toxicants can elicit distinct effects: low doses may sometimes induce a hormetic response, which is opposite to the destructive effects observed at high doses. Therefore, selecting an appropriate exposure dose is crucial. This study employed a dose of 150 mg/L, chosen to reflect realistic human exposure levels. Based on established scientific literature [55,56], we converted this dose for mice into a human-relevant equivalent using the body surface area conversion factor. This approach facilitates a more accurate assessment of the potential health impacts of DEHP on humans. By selecting a dose (150 mg/L) approximating human exposure levels, we were able to better investigate the effects of DEHP in mice and conduct a comprehensive assessment of its toxicity.

This study demonstrated that LBP treatment could significantly reverse the male reproductive toxicity induced by DEHP exposure and effectively alleviate adverse effects on sperm parameters, including reduced sperm count, abnormal motility parameters, and an increased rate of morphological abnormalities. Similar to our findings, numerous studies support the conclusion of this study. LBP has been shown to mitigate testicular damage and improve abnormal sperm parameters in diabetic animal models [57,58]. LBP has the potential to mitigate the cadmium exposure-induced decline in sperm motility and abnormal serum testosterone levels in mice [25]. LBP demonstrates the ability to counteract the doxorubicin-induced decline in testicular quality and enhance sperm concentration. Additionally, it exhibits reproductive protective effects through the reduction in lipid peroxidation and the promotion of testosterone synthesis [24]. In the bisphenol A exposure model, LBP pretreatment is capable of effectively restoring testicular mass, enhancing superoxide dismutase (SOD) activity, and reducing malondialdehyde (MDA) levels [59]. These studies conclusively demonstrate that LBP possesses the protective effect against testicular injury and enhances sperm motility. In this study, histopathological analysis revealed distinct pathological features in the testes of mice following DEHP exposure, including disordered arrangement of spermatogenic cells at all levels within the seminiferous tubules, a marked reduction in the number of spermatocytes, and vacuolation of Sertoli cells. In contrast, these pathological changes were significantly reversed in the LBP treatment group. This suggests that the protective effect of LBP may act on spermatocytes and Sertoli cells in the testis. Further RT-qPCR analysis revealed that DEHP exposure resulted in a significant down-regulation of mRNA expression levels of functional marker genes in Sertoli cells (*fshr*, *sox-9*, *abp*, and *ar*) as well as core regulatory genes involved in spermatocyte meiosis (*sycp3*, *stra8*, *vasa*, and *plzf*). Treatment with LBP was found to significantly restore the expression of genes associated with these functions. Similarly, LBP significantly alleviates the apoptosis of spermatogenic cells induced by low-dose ionizing radiation [26]. LBP can enhance the expression levels of ar, Occludin, and ZO-1 in heat stress-induced testicular Sertoli cells, thereby mitigating the damage to these cells caused by heat stress [60]. Therefore, our results indicate that LBP improves DEHP-induced testicular injury, possibly by protecting the functions of testicular spermatocytes and Sertoli cells.

Oxidative stress is intricately associated with male infertility [61]. Clinical studies indicate that approximately 80% of infertile men exhibit pathologically elevated levels of reactive oxygen species (ROS) in their semen. This condition has been clinically categorized as male oxidative stress-induced infertility (MOSI). It is important to highlight that exposure to DEHP may result in elevated oxidative stress levels in Sertoli cells and spermatocytes, thereby potentially impairing the functionality of spermatogenic cells [30,62]. Ferroptosis, as a form of iron-dependent programmed cell death, has a pathological mechanism that is closely associated with the imbalance of redox homeostasis and disorders in iron metabolism [63]. Previous studies have shown that LBP has the ability to resist oxidative stress and inhibit ferroptosis [28,35]. Therefore, this study investigated the mechanisms by which LBP modulates oxidative stress and ferroptosis in spermatocytes and Sertoli cells, ultimately ameliorating testicular injury induced by DEHP. As a critical antioxidant molecule, the depletion of GSH substantially impairs the oxidative stress defense capability of cells, leading to the abnormal accumulation of lipid peroxides such as MDA [38,64]. In this study, DEHP/MEHP exposure resulted in elevated MDA levels and reduced T-AOC and GSH levels in testicular tissue as well as in two cell lines (GC2 and TM4 cells). Notably, LBP intervention was found to effectively reverse these oxidative stress-related parameters. Similarly, LBP demonstrates antioxidant effects by suppressing ROS generation, decreasing MDA levels, and enhancing the enzymatic activities of superoxide dismutase (SOD) and glutathione peroxidase (GSH-Px) [65]. The occurrence of ferroptosis is intricately linked to the disruption of iron homeostasis in the context of iron metabolism regulation [66]. In this study, exposure to DEHP/MEHP was found to result in a significant increase in the concentration of free Fe^2+^ in testicular tissue, GC2 cells, and TM4 cells. Additionally, the expression levels of key proteins involved in iron homeostasis, including FTH1, TFR, and TF, were significantly up-regulated. Notably, LBP intervention demonstrated the ability to effectively reverse these abnormalities in iron metabolism indicators. A study has demonstrated that LBP pretreatment can significantly suppress the level of free Fe^2+^ as well as the expression of FTH1 [35]. Therefore, this study demonstrates that LBP effectively mitigates the ferroptosis process and exerts reproductive protective effects by restoring iron metabolic homeostasis in testicular spermatocytes and supporting cells, as well as inhibiting oxidative stress.

Interestingly, FTH levels were significantly elevated following DEHP treatment. This phenomenon may be attributed to a stress response to iron overload in cells under 150 mg/kg DEHP exposure. Consistent with our findings, elevated FTH1 was also observed in studies of PM2.5-induced ferroptosis in epithelial cells. This was ascribed to impaired cellular antioxidant capacity, leading to dysfunction in iron storage and ultimately triggering ferroptosis [67]. Furthermore, erastin (a ferroptosis inducer) also significantly up-regulated the mRNA levels of FTH1 and FTL [68]. Similarly, research by Li et al. demonstrated that silica nanoparticles (SiNPs) induced ferroptosis in cardiomyocytes both in vivo and in vitro, concurrently with an increase in FTH levels [69].

NRF2 serves as a critical regulatory hub for maintaining intracellular iron homeostasis and plays a central role by orchestrating the transcriptional network of genes involved in iron metabolism [39,40]. When cells are exposed to oxidative stress, NRF2 undergoes KEAP1-dependent dissociation and subsequent nuclear translocation. This process activates the transcription of downstream target genes by binding to Antioxidant Response Elements (AREs), including antioxidant-related genes such as the cystine/glutamate antiporter (SLC7A11, also known as xCT) and GPX4. Consequently, this mechanism helps maintain redox homeostasis [32,70]. Meanwhile, the light and heavy chains of ferritin, a key iron storage protein (FTL/FTH1), as well as ferroportin (FPN1), the iron transporter responsible for cellular iron efflux, are all transcriptionally regulated by NRF2 [71,72]. To investigate the mechanism by which NRF2 alleviates DEHP-induced ferroptosis in Sertoli cell and spermatocytes within the context of LBP, an intervention approach was employed involving the knockdown of NRF2 using siRNA and the application of the specific NRF2 inhibitor ML385. The findings demonstrated that suppression of NRF2 function not only exacerbated the substantial accumulation of intracellular Fe^2+^, but also resulted in elevated levels of MDA. These hallmarks of oxidative damage and iron homeostasis disruption collectively intensified the injury to both spermatocytes and Sertoli cells. Similar to this study, lignan ameliorates the pathological features of Alzheimer’s disease model mice by activating NRF2, thereby reducing oxidative stress and intracellular iron overload in brain tissue [73].

The novelty of this study lies in the concurrent verification of LBP’s antagonistic effects against the DEHP metabolite MEHP within both in vivo testicular tissues and the in vitro GC-2/TM4 cell model, thereby revealing LBP’s potential in mitigating reproductive injury induced by environmental toxins. However, limitations in the experimental design should be acknowledged. Primarily because the study lacked a dedicated LBP treatment group, it cannot definitively preclude LBP’s potential influence on the outcomes. Additionally, the underlying causes of aberrant FTH protein expression were not further explored, leaving its specific role and mechanism unelucidated. Furthermore, the research did not delve into the differences in toxicity mechanisms across various DEHP doses. To address these gaps, we plan to incorporate a dedicated LBP control group in future work, probe the specific role of FTH1 in ferroptosis, and establish a chronic exposure model employing environmentally relevant low doses to better approximate actual risk scenarios.

## 5. Conclusions

**Figure 7:**
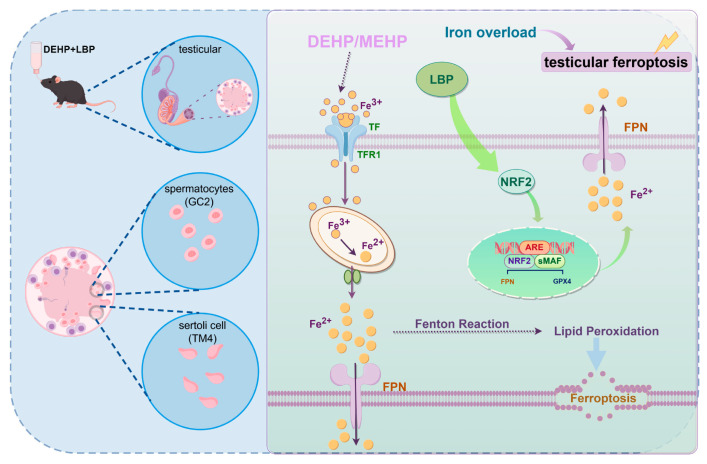
Mechanism diagram of LBP resisting DEHP-induced ferroptosis in testicular cells. MEHP induces abnormal accumulation of Fe^2+^ in Sertoli cells and spermatocytes, thereby triggering lipid peroxidation. LBP improves ferroptosis in spermatocytes and Sertoli cells by activating NRF2 to regulate the expression of iron homeostasis-related proteins. This figure was drawn with Figdraw.

Based on its findings, this study confirmed through both in vivo and in vitro experiments that LBP regulates cellular iron homeostasis and oxidative stress levels in Sertoli cells and spermatocytes by activating NRF2, thereby alleviating testicular injury induced by DEHP/MEHP. Specifically, LBP enhances intracellular iron excretion and effectively reduces the level of free Fe^2+^ by activating the transcriptional activity of NRF2 and its downstream target genes. Furthermore, the iron homeostasis regulated by LBP inhibits lipid peroxidation initiated by the Fenton reaction, ultimately resisting the ferroptosis process in Sertoli cells and spermatocytes (Figure 7). This study not only provides preventive strategies for DEHP-induced male reproductive injury but also validates the therapeutic potential of LBP as a food and medicine homologous substance, offering intervention strategies to maintain male reproductive health.

## Data Availability

Data will be made available on request.

## Supplementary Materials

The following supporting information can be downloaded at: https://www.mdpi.com/article/10.3390/toxics13080677/s1, Figure S1: LBP ameliorates DEHP-induced testicular injury and decline in sperm quality; Figure S2: LBP mitigates MEHP-induced ferroptosis in GC2 cells and TM4 cells through the NRF2/FPN1 pathway; Table S1: Sequences of oligonucleotide primers for QRT-PCR; Table S2: Antibody information in this study.
