# Supplementary material for: Lycium barbarum Polysaccharide Improves Iron Homeostasis in Spermatocytes and Sertoli Cells via NRF2 to Alleviate DEHP-Induced Male Reproductive Toxicity in Mice
Source: Toxics. 2025 Aug 14;13(8):677. doi: 10.3390/toxics13080677 (PMC12390077; doi:10.3390/toxics13080677)
Supplement: Supplementary file 1 [file toxics-13-00677-s001.zip › toxics-3786968-supplementary.pdf]

Supplemental Materials

***Lycium barbarum* polysaccharide improves iron homeostasis in spermatocytes and Sertoli cells via NRF2 to alleviate DEHP-induced male reproductive toxicity in mice**

Zhen Zhang, Yitong Shang, Hong Yang, Liyang Ding, Yu Deng,Bo Xu *, Xufeng Fu*

Key Laboratory of Fertility Preservation and Maintenance of Ministry of Education, School of Basic Medical Sciences, Ningxia Medical University, Yinchuan 750004, China

[1]Both authors (Zhen Zhang and Yitong Shang) contributed equally to this work and should be considered as equal first coauthors.

* Corresponding author. Key Laboratory of Fertility Preservation and Maintenance of Ministry of Education, School of Basic Medical Sciences, Ningxia Medical University, Yinchuan 750004, China. Email: xubonxmu@163.com(B. Xu); fuxufeng100@163.com (X. Fu); .

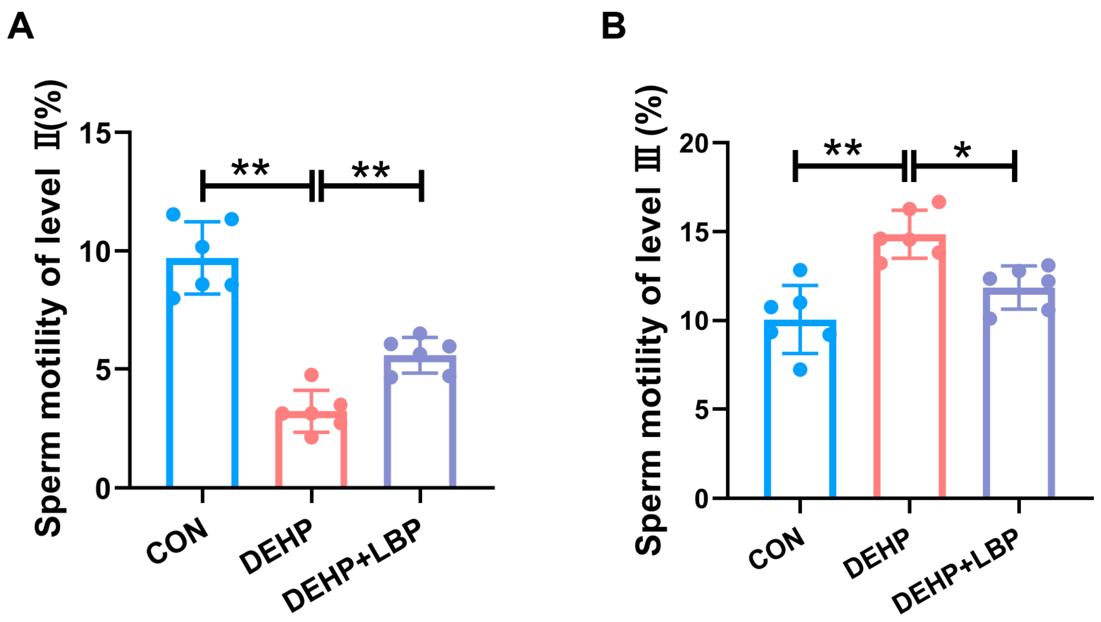

Figure S1. LBP ameliorates DEHP-induced testicular injury and decline in sperm quality. (A) Grade II sperm motility, with linear movement indicating good sperm motility. (B) Grade III sperm motility, with non-linear progressive movement representing moderate sperm motility.Data are presented as Mean ± SD. * $P < 0.05$, ** $P < 0.01$.

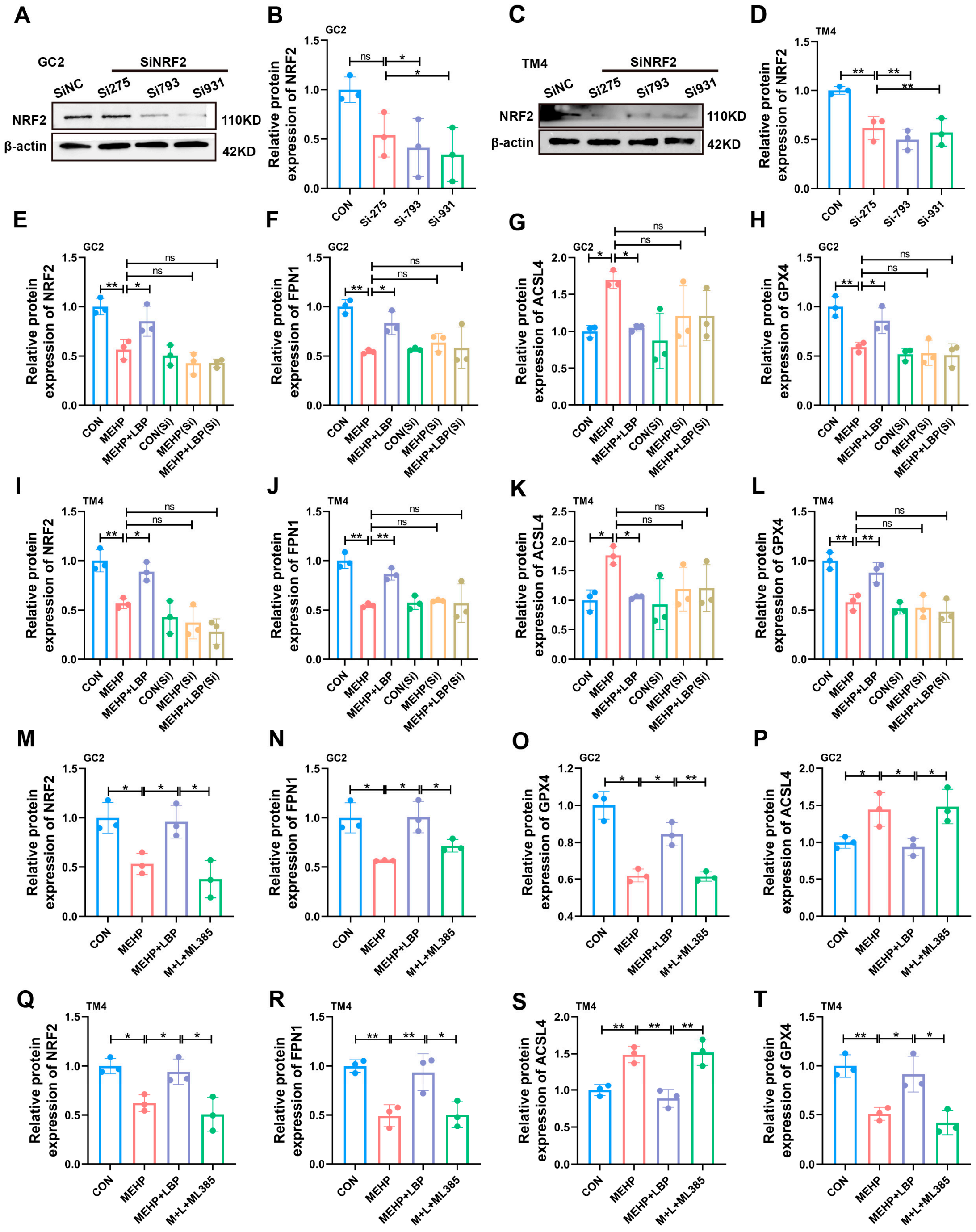

Figure S2. LBP mitigates MEHP-induced ferroptosis in GC2 cells and TM4 cells through the NRF2/FPN1 pathway. (A and C) NRF2 expression in the control group, MEHP group and MEHP+LBP group after NRF2 interference (n = 3). (E and H) Quantitative analysis of Fig. 6C. (I and L) Quantitative analysis of Fig. 6D. (M and P) Quantitative analysis of Fig. 6E. (Q and T) Quantitative analysis of Fig. 6F. Data are presented as Mean ± SD. * $P < 0.05$, ** $P < 0.01$. “ns” indicates non-significant differences.

Table S1. **Sequences of oligonucleotide primers for QRT-PCR.**

Gene Names	Sequence (5' → 3')	species
ACTIN	AGCCATGTACGTAGCCATCC<br>GCTGTGGTGGTGAAGCTGTA	Mouse
Ar	TCCAAGACCTATCGAGGAGCG<br>GTGGGCTTGAGGAGAACCAT	Mouse
Abp	GAAATCCAGCTACACAATGCC<br>AGCAGCAGTGAATCCCCGTT	Mouse
Fshr	TGCTCTAACAGGGTCTTCCTC<br>TCTCAGTTCAATGGCGTTCCG	Mouse
Sox-9	TCCCAAAACCGACGTGCAAG<br>TGCTCAGTTCACCGATGTCC	Mouse
Ptgs2	TGTGCGACATACTCAAGCAG<br>TGTTGCACGTAGTCTTCGAT	Mouse
Fth1	TGCCTCCTACGTCTATCTGTC<br>GCTTCATCAGTTTCTCGGCAT	Mouse
Slc7a11	CTATTTTACCACCATCAGTGCG<br>ATCGGGACTGCTAATGAGAATT	Mouse
Gpx4	ATAAGAACGGCTGCGTGGTGAAG<br>TAGAGATAGCACGGCAGGTCCTTC	Mouse
Tf	CCACTCAAATGTGCTCCGAAC<br>TTCCTGCTTCAGATTCTTAGCC	Mouse

Sycp3	GCTTCTTTCAAAGCCAGTAACC<br>CACTGCTGCAACACATTCATAA	Mouse
Stra8	C T C C T C C T C C A C T C T G T T G C<br>G C G G C A G A G A C A ATA G G A A G	Mouse
Vasa	CA GCTTCAG TA GCA GCA CAA G<br>CA T G A CTC G TC A TC A A CT G G A	Mouse
Dazl	ATGTTGTACCTCCGGCTTATTCA<br>CCA TTTCC A GA G G G T G G A G T A	Mouse
Plzf	G C A T T T A C T G G C T C A T T C A<br>G T A T G G G T C T G T C T G T G T	Mouse

Table S2. Antibody informations in this study.

Target	Vendor	Usage (Dilution)	attribute
GPX4	Abcam	WB (1:1000)	Rabbit
SLC7A11	Proteintech	WB (1:1000)	Rabbit
NRF2	Abcam	WB (1:1000)	Rabbit
PTGS2	Proteintech	WB (1:1000)	Rabbit
TF	Proteintech	WB (1:1000)	Rabbit
TFR	Abcam	WB (1:1000)	Rabbit
DMT1	Proteintech	WB (1:1000)	Rabbit
FTH1	invitrogen	WB (1:1000)	Rabbit
E-Cadherin	Cell signaling	WB (1:1000)	Rabbit
FSHR	Proteintech	WB (1:1000)	Rabbit
Vimentin	Cell signaling	WB (1:1000)	Rabbit
FPN1	Proteintech	WB (1:1000)	Rabbit
ZO-1	Bioss	WB (1:1000)	Rabbit
Occludin	Proteintech	WB (1:1000)	Rabbit
AR	Proteintech	WB (1:1000)	Rabbit
4-HNE	Abcam	IHC (1:200)	Rabbit

ACSL4	Abcam	WB (1:1000)	Rabbit
β-actin	servicebio	WB (1:1000)	Rabbit
GAPDH	servicebio	WB (1:1000)	Rabbit

WB: Western Blot. IHC: Immunohistochemistry
